# Surveillance of vector-borne pathogens under imperfect detection: lessons from Chagas disease risk (mis)measurement

**DOI:** 10.1038/s41598-017-18532-2

**Published:** 2018-01-09

**Authors:** Thaís Tâmara Castro Minuzzi-Souza, Nadjar Nitz, César Augusto Cuba Cuba, Luciana Hagström, Mariana Machado Hecht, Camila Santana, Marcelle Ribeiro, Tamires Emanuele Vital, Marcelo Santalucia, Monique Knox, Marcos Takashi Obara, Fernando Abad-Franch, Rodrigo Gurgel-Gonçalves

**Affiliations:** 1 Laboratório de Parasitologia Médica e Biologia de Vetores, Faculdade de Medicina, Universidade de Brasília, Brasília, 72910-900 Brazil; 2 Laboratório Interdisciplinar de Biociências, Faculdade de Medicina, Universidade de Brasília, Brasília, 72910-900 Brazil; 3 Laboratório Central de Saúde Pública, Secretaria Estadual de Saúde de Goiás, Goiânia, 74853-120 Brazil; 4 Diretoria de Vigilância Ambiental, Secretaria de Saúde do Distrito Federal, Brasília, 70086-900 Brazil; 5 Grupo Triatomíneos, Instituto René Rachou – Fiocruz, Belo Horizonte, 30190-009 Brazil

## Abstract

Vector-borne pathogens threaten human health worldwide. Despite their critical role in disease prevention, routine surveillance systems often rely on low-complexity pathogen detection tests of uncertain accuracy. In Chagas disease surveillance, optical microscopy (OM) is routinely used for detecting *Trypanosoma cruzi* in its vectors. Here, we use replicate *T*. *cruzi* detection data and hierarchical site-occupancy models to assess the reliability of OM-based *T*. *cruzi* surveillance while explicitly accounting for false-negative and false-positive results. We investigated 841 triatomines with OM slides (1194 fresh, 1192 Giemsa-stained) plus conventional (cPCR, 841 assays) and quantitative PCR (qPCR, 1682 assays). Detections were considered unambiguous only when parasitologists unmistakably identified *T*. *cruzi* in Giemsa-stained slides. qPCR was >99% sensitive and specific, whereas cPCR was ~100% specific but only ~55% sensitive. In routine surveillance, examination of a single OM slide per vector missed ~50–75% of infections and wrongly scored as infected ~7% of the bugs. qPCR-based and model-based infection frequency estimates were nearly three times higher, on average, than OM-based indices. We conclude that the risk of vector-borne Chagas disease may be substantially higher than routine surveillance data suggest. The hierarchical modelling approach we illustrate can help enhance vector-borne disease surveillance systems when pathogen detection is imperfect.

## Introduction

Vector-borne infectious diseases rank among the most relevant threats to public health globally^1^. Surveillance of pathogen presence in vectors allows epidemiologists to track variations of disease transmission risk in time and space. This, in turn, is crucial for the design, management, and evaluation of strategies for disease prevention^2–5^. To correctly interpret surveillance data, however, health officials need to understand how the tests used to ascertain vector infection actually perform. In particular, they need reliable estimates of each test’s sensitivity and specificity^6,7^. Sensitivity is defined in this context as the probability that the target pathogen is detected by a test, conditional on the vector being infected. Specificity is the probability that the pathogen is not detected by the test, conditional on the vector being uninfected. These two quantities are usually unknown and <100% for any given test or method – including, arguably, the best-studied diagnostic tests, whose nominal sensitivity and specificity are worked out under extremely artificial conditions that may be hard to replicate in field laboratories^8,9^.

Many tests are available for detecting pathogens in their hosts and vectors. They range from direct examination of samples under the microscope to sophisticated molecular assays that can detect minute amounts of the pathogen’s genetic material in tissue extracts^6,8–10^. The performance of these tests may vary substantially. This mainly depends on the balance between sensitivity and specificity – which as a rule trade-off against one another. Tests that have both high sensitivity and high specificity tend to be more costly, and often require higher-level skills, than simpler, yet not so well-performing, alternatives^8,10^. This is probably why many routine surveillance systems, particularly in developing countries, rely on low-complexity pathogen detection tests with suboptimal performance – they may be just the affordable and technically viable options^11,12^. Yet a suboptimal test will yield inaccurate data, and this measurement error can ultimately mislead decision makers^8,11^. In these cases, knowledge about test performance will provide particularly crucial insight into the performance of routine surveillance, thus widening the scope for sounder public health decision making. Still, widespread uncertainty about the true accuracy of pathogen detection tests in real-life surveillance settings substantially complicates any such assessment.

**Figure 1:**
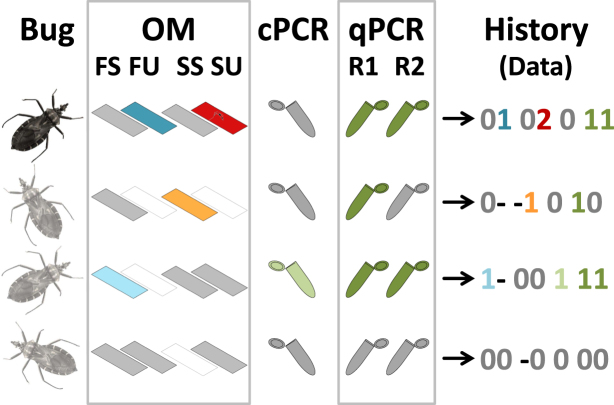
Detecting *Trypanosoma cruzi* in field-caught vectors. The figure illustrates our strategy of repeatedly checking for infection using (i) optical microscopy (OM) including slides read in routine surveillance (fresh, FS; Giemsa-stained, SS) or at the University of Brasília (fresh, FU; Giemsa-stained, SU), (ii) a conventional PCR (cPCR), and (ii) a replicate quantitative PCR (qPCR R1 and R2). Blank ‘slides’ represent OM slides that were not prepared for a given bug (coded ‘−’); in grey, tests that were scored as negative with ambiguity (possible false negatives, coded ‘0’); in light blue, dark blue, orange, light green, and dark green, tests scored as positive with ambiguity (possible false positives, coded ‘1’); and, in dark red with a parasite, a slide scored as positive without ambiguity (only when a professional parasitologists of the University of Brasília unmistakably identified *T*. *cruzi* trypomastigotes in a Giemsa-stained slide, coded ‘2’). The last column shows, for each bug, the “detection history” we used to construct our database, using the codes (‘−’, ‘0’, ‘1’, and ‘2’) defined above. Of the four bugs in this example, only the first one was scored as positive without ambiguity (hence its darker colour); the three light-coloured bugs might or might not have been infected: for the third and fourth, there were some ambiguous detections; for the last one, the six non-detections could have arisen either because the bug was not infected or because the tests failed to detect the parasite.

Here, we illustrate how a hierarchical modelling approach can help assess the performance of vector-borne disease surveillance in the face of imperfect pathogen detection. As a case-study, we investigate the detection of a major human parasite, *Trypanosoma cruzi*, in its insect vectors^13^. *T*. *cruzi* causes Chagas disease, one of the most important vector-borne diseases in the Americas^14^. The parasite is primarily transmitted by blood-sucking bugs known as triatomines, and entomological-parasitological routine surveillance (EPRS hereafter) is therefore a key component of Chagas disease control programs^13–16^. In most such programs, trained technicians identify suspect insects collected inside or around houses and check them for *T*. *cruzi* infection through optical microscopy (OM) of hindgut contents. OM-based detection of *T*. *cruzi*, however, is unlikely to be 100% sensitive or 100% specific^17–23^. *T*. *cruzi* surveillance data, then, likely contain a certain, yet largely unknown, amount of measurement error. To quantify this error, we applied multiple detection tests, from routine-surveillance OM to DNA-based methods, to over 800 field-caught vectors. We then used multiple detection-state site-occupancy models^24^ (a class of hierarchical models) to obtain statistical estimates of each test’s sensitivity and specificity while explicitly accounting for false-negative and false-positive results. These models require (i) replicate testing of a subset of the vectors and (ii) unambiguous identification of a subset of the infections (see Fig. 1 and ref.^24^). This strategy allowed us to compute corrected estimates of infection frequency in the five triatomine bug species most often found inside and around houses in central Brazil.

## Results

### Naïve infection indices

**Figure 2:**
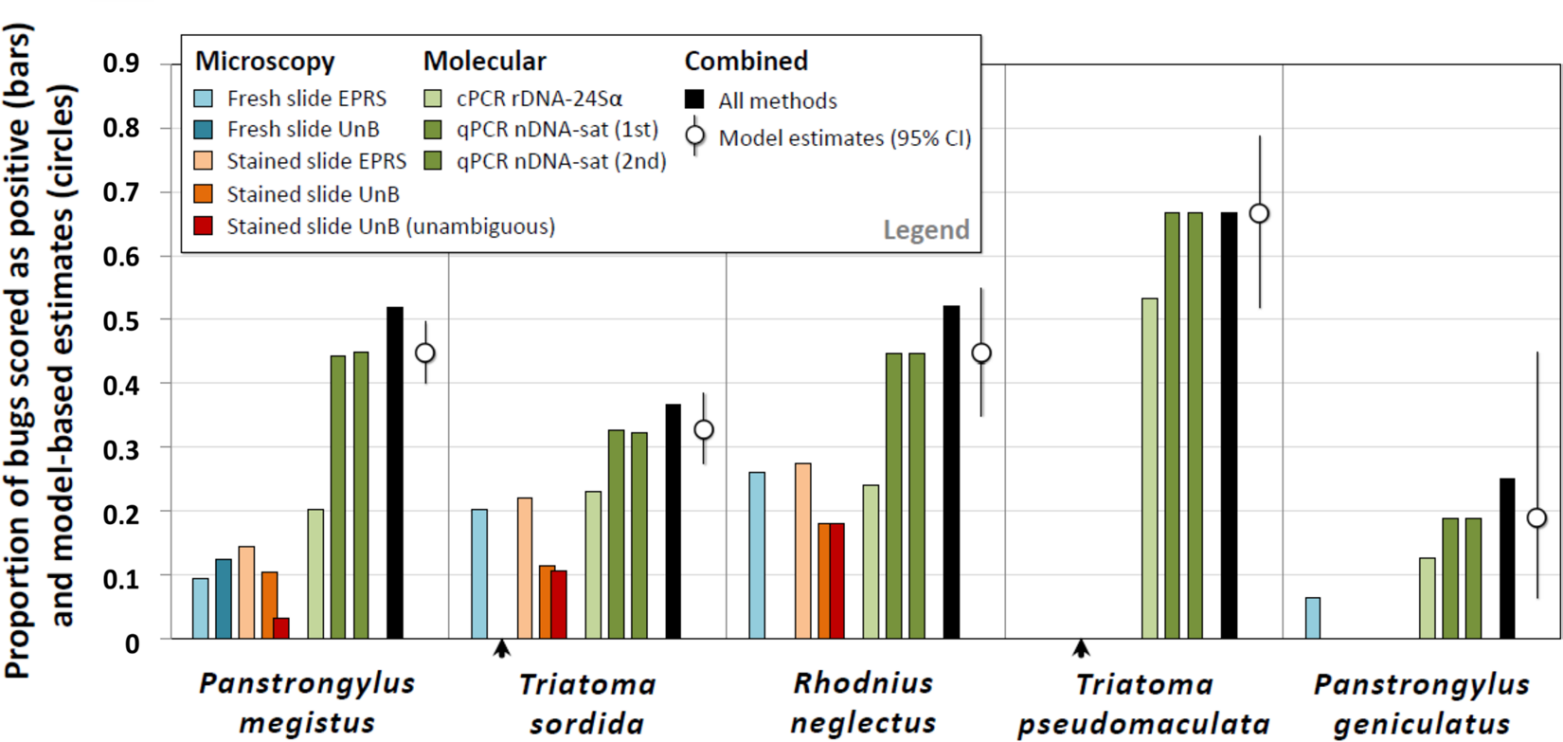
*Trypanosoma cruzi* infection in 841 triatomine bugs caught inside or around houses of central Brazil, 2012–2014. Bars represent the observed proportions of bugs scored as positive with different methods, and circles show infection probabilities as estimated by the focal site-occupancy model. EPRS, entomological-parasitological routine surveillance; UnB, University of Brasília; cPCR rDNA-24Sα, conventional PCR on the 24Sα subunit of the nuclear ribosomal DNA; qPCR nDNA-sat, quantitative PCR on the nuclear satellite DNA; CI, confidence interval; arrowheads indicate instances in which no bug was tested by a given method (see Table 2).

We investigated *T*. *cruzi* detection in 841 triatomine bugs of five species collected inside or around houses in the state of Goiás and the Federal District, Brazil. We used different combinations of OM slide readings (fresh and/or Giemsa-stained slides read in EPRS and/or at the University of Brasília [UnB]) and conventional plus real-time quantitative PCRs (cPCR and qPCR, respectively) (see Tables 1 and 2 and Methods). Overall, 397 bugs were scored as positive in at least one test, for a naïve infection index of 47.2%. Note, however, that this calculation relies on the assumption that the joint results of the tests applied to each bug ensure 100% sensitivity and 100% specificity. Test-specific naïve indices varied about two-fold, from 17.8% considering OM results only to 41.5% considering PCR results only – with 23.1% positive by cPCR and 41.3–41.4% by qPCR. Considering EPRS results only, infection with *T*. *cruzi* was reported in 15.1% of the bugs (from 0% to 27.2%, depending on species; Table 2, Fig. 2). As above, however, the direct interpretation of test-specific naïve values hinges on the assumption that each test, including OM-based detection of *T*. *cruzi* in EPRS, is 100% sensitive and 100% specific. Further descriptive details are presented in Tables 1 and 2 and in Fig. 2; the raw data are available in Supplementary Data S1, and PCR protocols in Supplementary Text S1.

**Table 1:** Optical microscopy (OM) slides examined to detect *Trypanosoma cruzi* infection in 841 triatomine bugs, central Brazil, 2012–2014. Fresh and Giemsa-stained slides were examined by entomological-parasitological routine surveillance (EPRS) and University of Brasília (UnB) staff in different combinations.

OM slide combinations	Fresh slides		Stained slides		Bugs^1^
	EPRS	UnB	EPRS	UnB	
Fresh EPRS + Fresh UnB	•	•			19
Fresh EPRS + Stained EPRS	•		•		29
Fresh EPRS + Stained UnB	•			•	67
Fresh UnB + Stained UnB		•		•	22
Fresh EPRS + Fresh UnB + Stained UnB	•	•		•	334
Fresh EPRS + Stained EPRS + Stained UnB	•		•	•	370
Total (slides/bugs)	819	375	399	793	(2386/841)

^1^All tested also by one conventional and two real-time quantitative PCRs. •Slide read.

**Table 2:** Numbers of triatomine bugs tested and scored as positive for *Trypanosoma cruzi* infection by optical microscopy and molecular methods.

Test		Triatomine bug species									
		*P*. *megistus*		*T*. *sordida*		*R*. *neglectus*		*T*. *pseudomaculata*		*P*. *geniculatus*	
		*n*	+	*n*	+	*n*	+	*n*	+	*n*	+
Fresh slide	EPRS	405	38	273	55	92	24	33	0	16	1
	UnB	332	41	0	−	1	0	40	0	2	0
Stained slide	EPRS	21	3	273	60	91	25	0	−	14	0
	UnB	395	41(12)	248	28(26)	89	16(16)	45	0(0)	16	0(0)
Microscopy^1^	EPRS	405	38	273	60	92	25	33	0	16	1
	UnB	414	42	248	28	89	16	45	0	16	0
	Total	415	64	273	60	92	25	45	0	16	1
cPCR		415	84	273	63	92	22	45	24	16	2
qPCR	1^st^	415	184	273	89	92	41	45	30	16	3
	2^nd^	415	186	273	88	92	41	45	30	16	3
Molecular^1^	Total	415	186	273	89	92	41	45	30	16	3
Total^1^		415	215	273	100	92	48	45	30	16	4

*P*., *Panstrongylus*; *T*., *Triatoma*; *R*., *Rhodnius*. EPRS, entomological-parasitological routine surveillance systems (state of Goiás and Federal District, Brazil); UnB, University of Brasília; cPCR, conventional PCR on the 24Sα subunit of the nuclear ribosomal DNA; qPCR, real-time PCR on nuclear satellite DNA For each species, the numbers of bugs tested (*n*) and scored as positive (+) are given; for Giemsa-stained microscope slides read at UnB, the number of bugs in which infection was unambiguously determined is also given (in parentheses). ^1^Bugs scored as positive in at least one test.

### Hierarchical modelling

**Figure 3:**
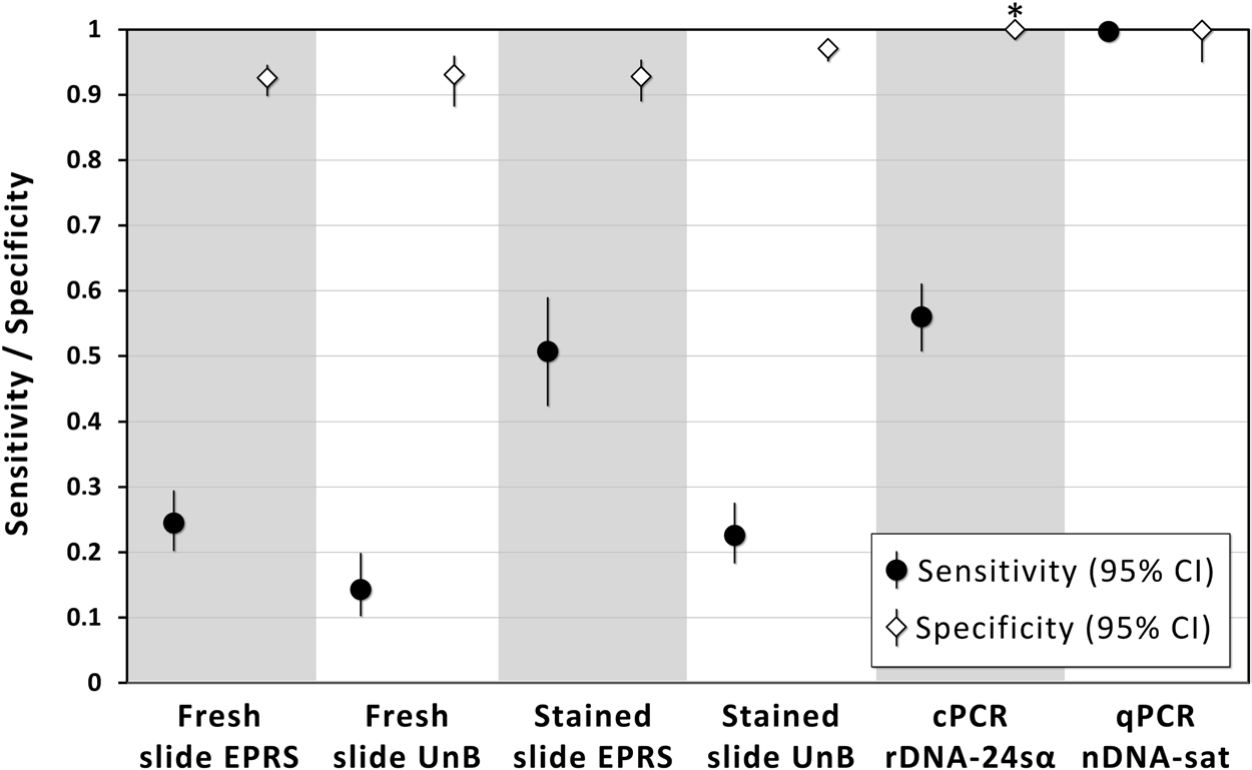
Performance of microscopy and PCR for the *Trypanosoma cruzi* detection in insect vectors: sensitivity and specificity as estimated through site-occupancy modelling. EPRS, entomological-parasitological routine surveillance; UnB, University of Brasília; cPCR rDNA-24Sα, conventional PCR on the 24Sα subunit of the nuclear ribosomal DNA; qPCR nDNA-sat, quantitative PCR on the nuclear satellite DNA; CI, 95% confidence interval (an asterisk indicates that the CI could not be estimated for cPCR specificity).

A site-occupancy model with bug species-specific infection probability and test-specific sensitivity and specificity (i.e., our focal model; see Methods) had a second-order Akaike’s Information Criterion (AICc) score^25^ from 18.5 to >1020 units smaller than models in which those parameters were held constant across, respectively, bug species and pathogen detection tests. These simpler models, hence, had no support from the data^25^, and we therefore base inference on our focal model. Figures 2 and 3 present, respectively, the estimates of species-specific infection frequency and of test-specific sensitivity and specificity computed using this model; the values of back-transformed estimates and 95% confidence intervals (CIs) are presented in Supplementary Table S1.

#### Test sensitivity and specificity

Our focal model suggests that sensitivity varies considerably, and specificity moderately, among tests (Fig. 3). The estimated sensitivity of OM-based tests was overall low, both in EPRS (fresh slide, 24.5%, CI 20.2–29.5%; stained slide, 50.7%, CI 42.4–59.0%) and at the UnB (fresh slide, 14.3%, CI 10.2–19.9%; stained slide, 22.6%, CI 18.3–27.6%). cPCR had low sensitivity (56.0%, CI 50.7–61.1%, comparable to that of Giemsa-stained OM slides read in EPRS), whereas qPCR had very high sensitivity (99.7%), with a lower CI limit just above 98% and an upper limit at 99.9% (Fig. 3 and Supplementary Table S1). Specificity was always estimated at > 90%, although 95% CIs overlapped that value for OM-based tests applied in EPRS (fresh slide, 92.6%, CI 89.8–94.6%; stained slide, 92.8%, CI 89.0–95.4%) and for fresh slides read at UnB (93.1%, CI 88.2–96.0%; stained slide, 97.1%, CI 95.1–98.3%). The estimate for cPCR specificity was virtually 1.0, with a large standard error suggesting that the maximum-likelihood solution lies close to the boundary. qPCR again performed very well, with an estimated specificity close to 100% and a lower CI limit of 95.0% (see Fig. 3 and Supplementary Table S1).

#### Frequency of infection in vectors

Along with the test performance estimates given above, our focal model yielded a corrected estimate of the frequency of *T*. *cruzi* infection (denoted Ψ) in each of the five vector species in our sample. As Fig. 2 and Table 2 and Supplementary Table S1 show, Ψ estimates were very close to naïve indices computed from qPCR data – which reflects the excellent performance of this test. Importantly, model-based infection estimates were consistently larger (about two times, from 1.3 to 3.0) than naïve indices computed from OM or cPCR (Fig. 2). Species-specific EPRS-based indices and model-based Ψ values compared as follows: *Panstrongylus megistus* 9.4% *vs*. 44.7%; *Triatoma sordida* 22.0% *vs*. 32.6%; *Rhodnius neglectus* 27.2% *vs*. 44.6%; *Triatoma pseudomaculata* 0% *vs*. 66.7%; and *Panstrongylus geniculatus* 6.3% *vs*. 18.8% (Fig. 2, Table 2). Model-based estimates were somewhat smaller than indices based on the combined results from all tests, reflecting the fact that OM-based tests likely yielded some false-positive results. Finally, the size of CIs around model-based Ψ estimates clearly highlights our uncertainty about infection probabilities when sample sizes are small – particularly for *P*. *geniculatus* (*n* = 16) but also, albeit to a lesser extent, for *T*. *pseudomaculata* (*n* = 45) and *R*. *neglectus* (*n* = 92) (see Fig. 2 and Supplementary Table S1).

## Discussion

In this study we have demonstrated the use of a hierarchical modelling approach^24^ to investigate the performance of routine surveillance in the context of a major vector-borne parasitic disease. The results show that detecting *T*. *cruzi* in its triatomine bug vectors can be difficult. The estimated sensitivity of OM-based tests was below 30% except for Giemsa-stained slides examined in EPRS – which, in any case, failed to detect about half of the infections (Figs 2 and 3). As a consequence, naïve indices based on EPRS data consistently underestimated the true frequency of *T*. *cruzi* infection in vectors caught inside or around houses in central Brazil. In our hands, cPCR of ribosomal DNA had low sensitivity (barely above 55%) but very high specificity (Fig. 3). The sensitivity and specificity of the more sophisticated qPCR of nuclear satellite DNA were both >99%; this, therefore, was the only test yielding nearly unbiased infection indices (Figs 2 and 3, Supplementary Table S1).

It is not surprising that detecting microscopic protozoa is a difficult task. Less widely realised, however, is the fact that imperfect detection can affect any organism including plants, insects, and up to large-sized mammals^26–30^. It also affects molecules, including antibodies or DNA^31–33^. The consequences of imperfect detection can be particularly problematic when the target organism is a human pathogen. In the clinical setting, efforts have concentrated on the development of better-performing diagnostic tests, but this usually implies higher costs and/or a need for more sophisticated equipment and skills. In disease surveillance, where large numbers of samples are often processed, such high-cost, high-technology tests are usually impractical. This is particularly evident in developing countries, where decentralised surveillance laboratories often lack the resources needed to use complex tests in routine practice. It should generally be feasible, however, to blindly re-examine a subset of samples with high-performance tests, such as qPCR, in central reference laboratories; these data could then be used to gauge the reliability of imperfect routine tests in an analytical framework similar to that described here^23,24,31,34–36^. This would be a fairly straightforward way to enhance disease surveillance when pathogen detection is imperfect. If, for example, a random subset of just 96 bugs from our sample were tested using just one high-performance qPCR assay (99% sensitive and 100% specific), mean EPRS slide-reading sensitivity would be estimated at 71.4% (CI 63.2–78.4) and specificity at 99.7% (CI 98.4–99.9). Despite a modest, yet evident, upward bias, these estimates may be seen as a potentially important improvement (particularly regarding sensitivity) over the typical stance of ignoring the problem by assuming that detection is always perfect (see Text S2).

It should be noted that in some Chagas disease EPRS systems each bug is examined twice – one with a fresh slide and one with a Giemsa-stained slide. According to the estimates from our focal, top-ranking model, and assuming independence, the joint sensitivity of a two-slide test would be *Se*_joint_ = 1 − (1 − *Se*_fresh_) × (1 − *Se*_stained_) = 1 − (1–0.245) × (1–0.507) = 0.628, and the joint specificity *Sp*_joint_ = 1 − (1 − (*Sp*_fresh_ × *Sp*_stained_)) = 0.859 (see Supplementary Table S1 for estimate values). This suggests that, on average, such a two-slide–reading tactic would miss about 37% of infections and would mistakenly score as positive about 14% of the bugs. Albeit somewhat disappointing, these figures do not look entirely hopeless; they suggest that increasing slide-reading sensitivity might be worth the effort: if, for example, the very low sensitivity of fresh-slide reading could be raised to about 50%, then joint sensitivity would be ~75% and only ~25% of infections would be missed. This could perhaps be achieved by simply increasing the number of fresh-slide fields examined in EPRS.

**Figure 4:**
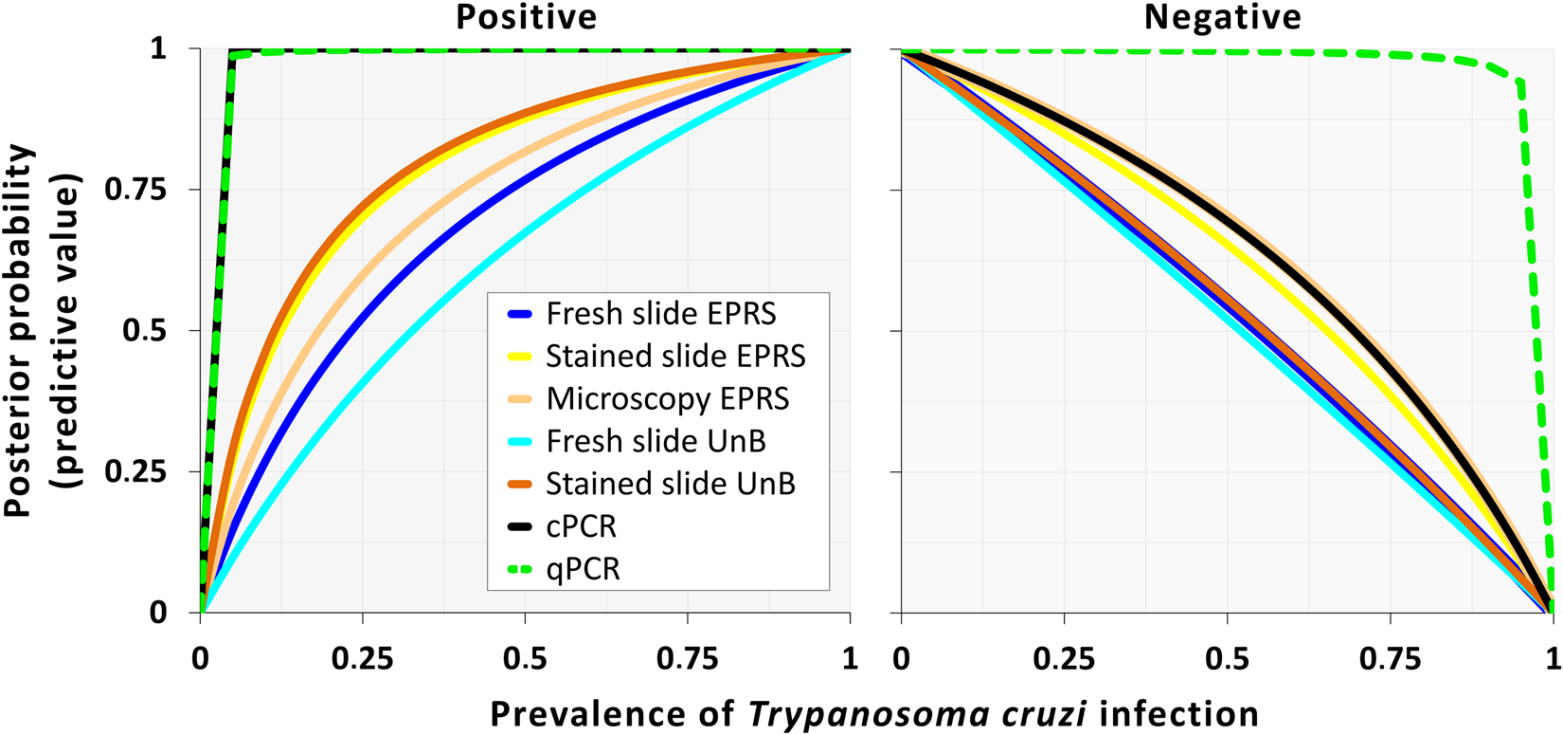
Posterior probabilities of vector infection (or non-infection) with *Trypanosoma cruzi*, given test results: positive and negative predictive values. EPRS, entomological-parasitological routine surveillance; UnB, University of Brasília; cPCR, conventional PCR on the 24Sα subunit of the nuclear ribosomal DNA; qPCR, quantitative PCR on the nuclear satellite DNA. Note that positive predictive values of cPCR (black line) and qPCR (green broken line) largely overlap.

Once reliable estimates of test sensitivity and specificity have been obtained, one can use Bayes’ theorem to calculate posterior probabilities of infection (or non-infection), given test results^37^. The posterior probability of infection, given the bug was scored as positive in a test, is also known as the positive predictive value of that test, and the posterior probability of non-infection, given a negative test result, as the negative predictive value^7^. These values depend not only on test performance, but also on the true prevalence of infection in the population the specimen was drawn from^7,37^. Using test performance estimates from our focal model (including joint performance of two OM slides read in EPRS as given above), and assuming each bug was randomly sampled from the population it belonged to, we calculated the positive and negative predictive values of each test at prior prevalence ranging from 0 to 100%. Figure 4 shows the obviously better performance of qPCR relative to all other tests; although cPCR also yields very good positive predictive values, a negative result is not very reliable as an indicator of absence of infection – except, as with the other tests, when the prevalence in the population is very low (Fig. 4). The graph also highlights the relatively poor performance of OM tests as used in EPRS (Fig. 4).

A reliable surveillance system for any vector-borne infection, including Chagas disease, has two main components. Entomological-parasitological surveillance aims at providing data on how often the vectors, and in particular infected vectors, are found in close proximity to humans^2–5,14,15^. Epidemiological surveillance aims at detecting human disease cases – and, in particular, new cases, which provide insight on incidence^3,4,38^. Here we have shown that EPRS data are likely to substantially underestimate natural *T*. *cruzi* infection in Chagas disease vectors. We know, in addition, that detecting infestation by those vectors can also be difficult, with sensitivity estimates often below 50%^27,28,39^. Finally, Chagas disease patients typically present with few signs and symptoms, if any, in the acute phase of infection, and it is estimated that less than 5% of new cases are diagnosed^14^. Chagas disease routine surveillance data therefore contain three types of (downward) measurement error – infestation by triatomines, vector infection, and human disease all are almost surely more frequent than reported^39^. This suggests that the risk of Chagas disease transmission to humans is almost surely higher than what crude surveillance data would seem to imply. If unaccounted for when interpreting such data, this composite error can generate a false sense of security that may ultimately mislead expert advisors and decision makers into, respectively, the wrong advice and the wrong decisions^39^.

Our results come with some important caveats; most of them relate to model assumptions, as outlined in the Methods section. We caution, in particular, that the conditional independence assumption common to most diagnostic test evaluations (e.g., those based on standard latent class analysis^23,34,40,41^) may not fully hold in our data. This means that our estimates of test performance metrics may be somewhat optimistic, which would lead to some bias in infection frequency estimates – likely a downward bias for the higher estimates and an upward bias for the lower estimates^40^. Our approach, on the other hand, let us relax the assumption of perfect detection and hence circumvent the need for internal amplification controls in PCR assays – whose absence is a widespread, major limitation of studies that assume perfect detection^42^. We also stress that OM examination of fresh slides is seldom meant to identify *T*. *cruzi* parasites; instead, the examiner usually records the detection of motile microorganisms and labels them as ‘trypanosomatids’. This is typically regarded as an initial, supposedly more sensitive test whose results need to be confirmed by the supposedly more specific examination of a stained slide^14^. In a sense, then, our estimates of fresh-slide specificity are somewhat ‘unfair’. We found, however, very small differences in specificity for fresh and stained slides (Fig. 3), suggesting that a similar, small fraction of fresh and stained slides scored as positive did not contain viable *T*. *cruzi* parasites. Further analyses (OM- and PCR-based) at UnB revealed *Trypanosoma rangeli* in seven and *Blastocrithidia triatomae* in 85 of the bugs in our sample^43^; these results will be presented elsewhere. Finally, we note that we formally considered only a few among the many potential sources of heterogeneity in test performance. In particular for OM, the skills of staff preparing and reading slides, their adherence to written protocols, the quality of reagents and microscopes, or bug-specific parasite loads may all vary and have an effect on performance. We did not have the means to measure or model all these plausible candidate predictors; instead, we present average test performance estimates for what may be considered typical circumstances of EPRS.

In summary, we have demonstrated how hierarchical site-occupancy models can help us develop a more realistic understanding of the performance of *T*. *cruzi* detection tests – from the OM-based methods widely used in routine surveillance to sophisticated molecular assays. This quantitative knowledge about test performance allowed us to compute corrected estimates of the frequency of *T*. *cruzi* infection in the five vector species most often found in houses across central Brazil. Our analyses revealed a considerable downward bias in *T*. *cruzi* infection indices generated by routine surveillance; since vector occurrence indices are also probably biased low, we conclude that the frequency at which infected triatomines occur inside or around houses, and hence the risk of Chagas disease transmission, may be substantially higher than surveillance data suggest. It is rather likely that similar biases affect routine surveillance systems aimed at other pathogens and other vectors. Any such bias must be singled out, quantified, and explicitly taken into account if we are to draw sound, epidemiologically meaningful conclusions from imperfect surveillance data.

## Methods

### The bugs

From August 2012 to December 2014, the parasitology laboratory at UnB received 841 triatomine bugs caught inside or around houses during EPRS in Goiás and the Federal District of Brazil. All bugs were identified to species at UnB using the keys by Lent & Wygodzinsky^13^.

### Optical microscopy

EPRS staff checked 420 bugs for *T*. *cruzi* infection with one fresh OM slide only and 399 bugs with two slides – one fresh and one Giemsa-stained (see below). The results of each OM examination carried out in EPRS, as well as stained slides, were sent together with the bugs to the UnB, where we examined 456 bugs with one OM slide only (19 fresh only, 437 stained only) and 356 bugs with two OM slides (one fresh, one stained). At the UnB, each slide was read for up to 5 minutes; if slim trypomastigotes with a large, round, subterminal kinetoplast^44,45^ were seen in a slide, then it was scored as unambiguously positive (see below) and the examination stopped. Table 1 shows the number of bugs examined with different combinations of OM slides both in EPRS and at UnB. Each fresh slide was prepared by homogenizing one droplet of bug hindgut contents (faeces and possibly urine) in one drop (~50 μL) of buffered saline solution on a microscope slide and covering it with a cover slip; the slide was examined under a light microscope at 400× magnification^17,22,44^. For Giemsa staining, thin smears of hindgut contents homogenised in saline solution were fixed with methanol and stained with a buffered 10% Giemsa stain solution; these slides were examined at 1000× magnification^44,45^. Fresh slides were scored as positive whenever motile forms suggestive of *T*. *cruzi* infection were observed; fresh slide scoring was always considered ambiguous. Stained slides were scored as unambiguously positive only when parasites were unmistakably identified as *T*. *cruzi* by UnB researchers; in all other cases, the results were considered ambiguous (see *Identifying and coding ambiguity* below).

### DNA-based methods

We stored all bugs at −20 °C at UnB. The bugs were thawed and dissected on sterilised glass slides in a laminar flow safety cabinet. The hindgut was removed with watchmaker forceps and stored in sterile phosphate-buffered saline at −20 °C until DNA extraction. The forceps were thoroughly washed twice (with HCl 0.1 M and with 70% ethanol) and flamed before re-use. DNA was extracted with the Illustra Tissue and Cells Genomic kit (GE Healthcare, Piscataway, NJ), the QIAamp DNA Mini Kit (Qiagen, Valencia, CA), or the Biopur Mini Spin Plus kit (Biometrix, Curitiba, Brazil) according to each manufacturer’s instructions. DNA was quantified with a NanoVue Plus spectrophotometer (GE). We used the QIAamp DNA Mini Kit to extract DNA from (i) one *T*. *cruzi* culture (Berenice strain, 5 × 10^5^ epimastigotes/mL) as the positive control and (ii) the hindguts of laboratory-reared, uninfected triatomines (*Dipetalogaster maxima*) as the negative control. Positive, negative, and blank controls (with no DNA) were included in each PCR round. To ensure that all DNA extracts from bug hindguts contained good-quality DNA, we PCR-amplified ~414 bp of the bugs’ mitochondrial cytochrome *b* gene^46^, including blank controls in each PCR run (Supplementary Text S1). Each bug was tested for *T*. *cruzi* DNA using two different PCRs. First, we ran a cPCR that targets the variable D7 domain of the 24Sα gene of the nuclear ribosomal DNA^47^. A bug was scored as cPCR-positive (with ambiguity; see below) when the assay yielded a 270–290-bp band^47^. Second, we ran a qPCR targeting the nuclear repetitive satellite region of *T*. *cruzi* ^48^ and using SYBR Green technology^49^. For each bug, we ran two independent qPCR assays. To score detection/non-detection of the parasite’s DNA by qPCR, we built a standard curve^50^ with serial dilutions of *T*. *cruzi* DNA extracted from an epimastigote culture (see above) and ranging from 10^−1^ to 10^4^ parasite equivalents/mL. As per standard curve results, a bug was scored as qPCR-positive (with ambiguity; see below) when the assay yielded a signal corresponding to ≥ 0.1 parasites/mL (see Supplementary Text S1). We note that all samples contained good-quality DNA (as indicated by amplification of the ~414-bp cytochrome *b* fragment) and all positive, negative, and blank controls yielded the expected results.

### Modelling

To assess how different tests, including those used in EPRS, perform at detecting *T*. *cruzi* infection in triatomine bugs, we used the hierarchical modelling approach developed by Miller *et al*.^24^. These ‘multiple detection-state site-occupancy models’ explicitly accommodate both false-negative and false-positive results. The models make use of repeated detection/non-detection data (possibly with missing results) to compute maximum-likelihood estimates of (i) the probability that an organism (here, *T*. *cruzi*) is detected in a sampling unit where it actually occurs (here, a *T*. *cruzi*-infected bug), and (ii) the probability that the organism is detected in a sampling unit where, in reality, it does not occur, i.e., the false-positive error rate^24^. With this information, the latent, unobserved probability of each sampling unit being ‘occupied’ by the target organism (here, probability of infection) is also estimated^24^. These probabilities can in addition be modelled as a function of covariates in a generalised linear modelling framework^24^. The models require that at least a subset of sampling units is checked for the presence of the target organism more than once, and that a subset of the detections can be considered unambiguous^24^. Below we give details on the modelling approach, which we implemented in Presence 11.8 (ref.^51^).

#### Identifying and coding ambiguity

We considered detection of *T*. *cruzi* as unambiguous (coded ‘2’) only when *T*. *cruzi* parasites with normal morphology (slim trypomastigotes with a large, round, subterminal kinetoplast – which were judged viable) were unmistakably identified in Giemsa-stained OM slides examined at UnB^45,52–54^. For the rest of tests and trials, all detections and non-detections were considered ambiguous and coded ‘1’ and ‘0’, respectively. We thus explicitly acknowledge the possibility that some positive results may be false positives and some negative results false negatives^24^. Microscope slide examination often yields false-negative results^17–23^. False positives may occur because *T*. *cruzi* and other triatomine-infecting trypanosomatids, such as *Blastocrithidia* spp. or *T*. *rangeli*, can be mistaken for one another, especially in fresh OM slides^52,54^. In the case of PCRs, cross-amplification of heterospecific genomic targets may produce false-positive detections. In addition, a sample containing *T*. *cruzi* DNA but no viable parasites may yield a positive, yet epidemiologically irrelevant, PCR result. Cross-sample spillover of amplicons (i.e., contamination), another source of false-positive PCRs, is routinely minimised with appropriate protocols for handling samples and can be detected using negative controls. Finally, polymerase inhibition or variation in primer-binding sequences may yield false-negative PCR results. To account for this pervasive potential for ambiguity, when specifying our models we fixed at 0 the probability (denoted *b*) that a detection event was classified as unambiguous, given the bug was infected and a detection occurred, for all tests except stained OM slides read at UnB – for which *b* was estimated.

#### Defining outcomes and modelling effects

We defined sensitivity (denoted *Se*) as the probability of detecting viable *T*. *cruzi* parasites, conditioned on their occurrence, in a given bug sample. We let *Se* vary as a function of detection test. Each test was also allowed to have its own probability of false-positive detections, denoted *P*_false_ (with 1 − *P*_false_ estimating the specificity *Sp* of each test). As noted above, *b* values were only estimated for stained OM slides read at UnB. In our research setting, ‘site-occupancy’ corresponds to the frequency of *T*. *cruzi* infection in vectors – the probability Ψ that a bug is ‘occupied by’ (i.e., infected with) viable *T*. *cruzi* parasites. To get species-specific estimates of this probability, we let Ψ vary among triatomine bug species. We then computed maximum-likelihood estimates of the parameters and effects of interest (and their variances) using Miller *et al*.’s^24^ models and Presence 11.8^51^. We compared our focal model (estimating test-specific *Se* and *Sp* and species-specific Ψ) with simpler models assuming constant Ψ across triatomine species or constant *Se* and *Sp* across tests. We used Akaike’s Information Criterion scores corrected for finite sample size (AICc, with *N* = 841) to evaluate relative model performance^25^.

#### Model assumptions

As in previous applications of the site-occupancy approach^24,31–33,35,36^ and other forms of latent class analysis^23,34^, our models assume conditional independence of diagnostic test results. This means that, conditional on the true infection status of a bug, whether one test yields the right (or wrong) result does not affect the probability that another test will also yield the right (or wrong) result^40^. If there is positive covariance between test results, then false-positive and false-negative error rate estimates will be too low and sensitivity and specificity will be overestimated; infection frequency estimates might also be somewhat biased high for bug species with lower infection frequency and somewhat biased low for bug species with higher infection frequency^40^. The models also assume independence of individual bugs in relation to infection status, equal test performance across bug species, and that the infection status of the bugs does not change between OM and DNA extraction^24^. Because we apply multiple tests with two distinct biological underpinnings to a large sample of bugs in several species, we believe that our estimates are unlikely to be badly biased and their confidence intervals unlikely to be exceedingly narrow. We stress, however, that the possible effects of violation of the assumptions above must be kept in mind when interpreting our results.

## Electronic supplementary material

Supplementary information

Supplementary Data S1
