# Author Correction: Surveillance of vector-borne pathogens under imperfect detection: lessons from Chagas disease risk (mis)measurement

**DOI:** 10.1038/s41598-018-24849-3

**Published:** 2018-05-04

**Authors:** Thaís Tâmara Castro Minuzzi-Souza, Nadjar Nitz, César Augusto Cuba Cuba, Luciana Hagström, Mariana Machado Hecht, Camila Santana, Marcelle Ribeiro, Tamires Emanuele Vital, Marcelo Santalucia, Monique Knox, Marcos Takashi Obara, Fernando Abad-Franch, Rodrigo Gurgel-Gonçalves

**Affiliations:** 1 Laboratório de Parasitologia Médica e Biologia de Vetores, Faculdade de Medicina, Universidade de Brasília, Brasília, 72910-900 Brazil; 2 Laboratório Interdisciplinar de Biociências, Faculdade de Medicina, Universidade de Brasília, Brasília, 72910-900 Brazil; 3 Laboratório Central de Saúde Pública, Secretaria Estadual de Saúde de Goiás, Goiânia, 74853-120 Brazil; 4 Diretoria de Vigilância Ambiental, Secretaria de Saúde do Distrito Federal, Brasília, 70086-900 Brazil; 5 Grupo Triatomíneos, Instituto René Rachou – Fiocruz, Belo Horizonte, 30190-009 Brazil

Correction to: *Scientific Reports* 10.1038/s41598-017-18532-2, published online 09 January 2018

This Article contains an error in Figure 1, in which the colours of certain slides are incorrect. The correct Figure 1 appears below.

**Figure 1:**
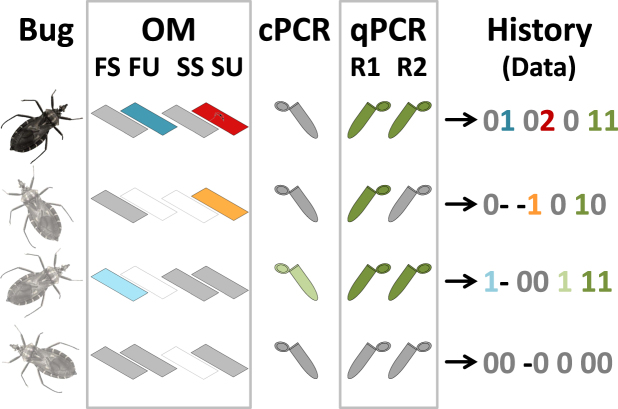
Detecting *Trypanosoma cruzi* in field-caught vectors. The figure illustrates our strategy of repeatedly checking for infection using (i) optical microscopy (OM) including slides read in routine surveillance (fresh, FS; Giemsa-stained, SS) or at the University of Brasília (fresh, FU; Giemsa-stained, SU), (ii) a conventional PCR (cPCR), and (ii) a replicate quantitative PCR (qPCR R1 and R2). Blank ‘slides’ represent OM slides that were not prepared for a given bug (coded ‘−’); in grey, tests that were scored as negative with ambiguity (possible false negatives, coded ‘0’); in light blue, dark blue, orange, light green, and dark green, tests scored as positive with ambiguity (possible false positives, coded ‘1’); and, in dark red with a parasite, a slide scored as positive without ambiguity (only when a professional parasitologists of the University of Brasília unmistakably identified *T*. *cruzi* trypomastigotes in a Giemsa-stained slide, coded ‘2’). The last column shows, for each bug, the “detection history” we used to construct our database, using the codes (‘−’, ‘0’, ‘1’, and ‘2’) defined above. Of the four bugs in this example, only the first one was scored as positive without ambiguity (hence its darker colour); the three light-coloured bugs might or might not have been infected: for the second and third, there were some ambiguous detections; for the last one, the six non-detections could have arisen either because the bug was not infected or because the tests failed to detect the parasite.

In addition, the Article contains an error in the legend of Figure 1.

“Of the four bugs in this example, only the first one was scored as positive without ambiguity (hence its darker colour); the three light-coloured bugs might or might not have been infected: for the third and fourth, there were some ambiguous detections; for the last one, the six non-detections could have arisen either because the bug was not infected or because the tests failed to detect the parasite.”

should read:

“Of the four bugs in this example, only the first one was scored as positive without ambiguity (hence its darker colour); the three light-coloured bugs might or might not have been infected: for the second and third, there were some ambiguous detections; for the last one, the six non-detections could have arisen either because the bug was not infected or because the tests failed to detect the parasite.”

This Article also contains an error in the Acknowledgements section.

“We thank the vector and parasite surveillance staff of Goiás State Health Department and the Federal District Environmental Surveillance Agency, Brazil. We also thank F. das Chagas, D.A. Rocha, and V.J. de Mendonça for assistance. M.R.F. de Oliveira made useful comments on an earlier draft of the manuscript, and R.N. This work was funded by the Coordenação de Aperfeiçoamento de Pessoal de Nível Superior (CAPES, grant 1276/2011) and the Fundação de Amparo à Pesquisa do Distrito Federal (FAP-DF, grant 6098/2013), Brazil. Additional support came from the Instituto René Rachou and the Vice-Presidência de Pesquisa e Laboratórios de Referência (both at Fiocruz, Brazil).”

should read:

“We thank the vector and parasite surveillance staff of Goiás State Health Department and the Federal District Environmental Surveillance Agency, Brazil. We also thank F. das Chagas, D.A. Rocha, and V.J. de Mendonça for assistance. M.R.F. de Oliveira made useful comments on an earlier draft of the manuscript. This work was funded by the Coordenação de Aperfeiçoamento de Pessoal de Nível Superior (CAPES, grant 1276/2011) and the Fundação de Amparo à Pesquisa do Distrito Federal (FAP-DF, grant 6098/2013), Brazil. Additional support came from the Instituto René Rachou and the Vice-Presidência de Pesquisa e Laboratórios de Referência (both at Fiocruz, Brazil).”

