# Supplementary material for: Surveillance of vector-borne pathogens under imperfect detection: lessons from Chagas disease risk (mis)measurement
Source: Sci Rep. 2018 Jan 9;8:151. doi: 10.1038/s41598-017-18532-2 (PMC5760667; doi:10.1038/s41598-017-18532-2)
Supplement: Supplementary file 1 — Supplementary information [file 41598_2017_18532_MOESM1_ESM.pdf]

**Supplementary information**

Title:

**Surveillance of vector-borne pathogens under imperfect detection:**

**lessons from Chagas disease risk (mis)measurement**


Authors:

Thaís Tâmara Castro Minuzzi-Souza, Nadjar Nitz, César Augusto Cuba Cuba, Luciana Hagström, Mariana Machado Hecht, Camila Santana, Marcelle Ribeiro, Tamires Emanuele Vital, Marcelo Santalucia, Monique Knox, Marcos Takashi Obara, Fernando Abad-Franch, Rodrigo Gurgel-Gonçalves


## Text S1 – PCR protocols

### 1. Triatomine bug mitochondrial cytochrome *b* (414bp)

*Primers* (Lyman et al., 1999):

- CytBF 5'GGACAAATATCATTTTGAGGAGCAACAG
- CytBR 5'ATTACTCCTCCTAGCTTATTAGGAATTG

*Reaction mix* (final volume 25 µl): 1x PCR reaction buffer (20 mM Tris-HCl pH 8.4, 50 mM KCl; Invitrogen), $MgCl_2$ 2.0 mM, dNTPs 0.2 mM (Illustra, GE), 0.2 µM of each primer, 1.5 U Taq Polymerase Platinum (Invitrogen), 4 µl DNA template.

*Equipment*: BIO-RAD MyCycler (Bio-Rad Laboratories).

*Thermal cycling*: 94°C 5 min, 35 cycles [94°C 45 sec, 50°C 45 sec, 72°C 1 min], 72°C 7 min, 4°C indefinite.

*Visualization/scoring*: electrophoresis in agarose gel 1.3% with ethidium bromide 0.5 mg/ml in TAE buffer (Tris-acetate 90 mM pH 8.0, EDTA 25 mM); UV transilluminator plus image capture with the AlphaImager Mini System (Alpha Innotech).

### 2. *Trypanosoma cruzi* conventional PCR (cPCR): nuclear ribosomal DNA (rDNA-24sα; 270–290bp)

*Primers* (Schijman et al., 2006):

- D75 5'GCAGATCTTGGTTGGCGTAG
- D76 5'GGTTCTCTGTTGCCCCTTTT

*Reaction mix* (final volume 25 µl): 1x PCR reaction buffer (20 mM Tris-HCl pH 8.4, 50 mM KCl; Invitrogen), $MgCl_2$ 1.5 mM, dNTPs 0.2 mM (Illustra, GE), 0.4 µM of each primer, 1.5 U Taq Polymerase Platinum (Invitrogen), 4 µl DNA template.

*Equipment*: BIO-RAD MyCycler (Bio-Rad Laboratories).

*Thermal cycling*: 94°C 3 min, 4 rounds of 3 cycles each [94°C 1 min, round-specific annealing (62°C/60°C/58°C/56°C) 1 min, 72°C 1 min], 35 cycles [94°C 1 min, 52°C 1 min, 72°C 1 min], 72°C 10 min, 4°C indefinite.

*Visualization/scoring*: electrophoresis in agarose gel 2% with ethidium bromide 0.5 mg/ml in TAE buffer (Tris-acetate 90 mM pH 8.0, EDTA 25 mM); UV transilluminator plus image capture with the AlphaImager Mini System (Alpha Innotech).

**3. *Trypanosoma cruzi* real-time quantitative PCR (qPCR): nuclear repetitive satellite region** (nDNA-sat)

*Primers* (Ndao et al., 2000):

- $TCRUZ_1$ 5’TGCACTCGGCTGATCGTTT
- $TCRUZ_2$ 5’ATTCCTCCAAGCAGCGGATA

*Reaction mix* (final volume 20 µl): 1x Power SYBR Green PCR Master Mix (Applied Biosystems), 0.2 µM of each primer, 2 µl DNA template (10 ng).

*Equipment*: StepOnePlus Real Time PCR System (Applied Biosystems).

*Thermal cycling*: carried out in duplicate in 96-well plates (MicroAmp) and 50°C 2 min, 95°C 10 min, 40 cycles [95°C 15 sec, 60°C 45 sec, 72°C 10 sec].

*Visualization/scoring*: StepOne v 2.3 software (Applied Biosystems).

*Standard curve*:

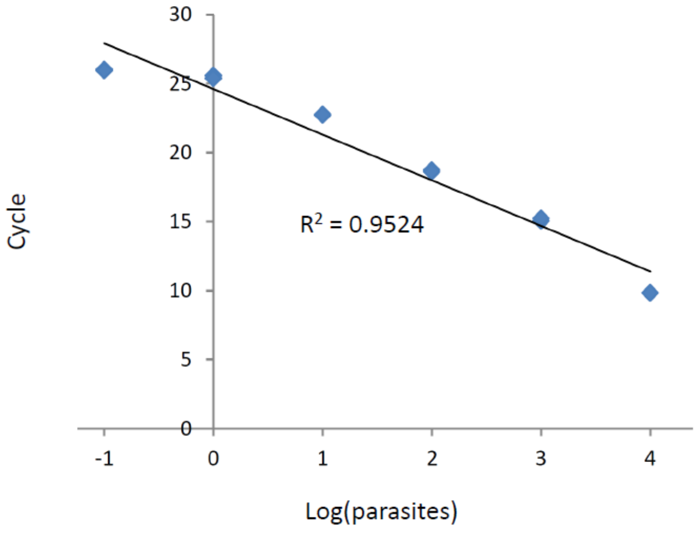

## References

Lyman, D.F. *et al*. Mitochondrial DNA sequence variation among triatomine vectors of Chagas' disease. *Am. J. Trop. Med. Hyg.* **60**, 377-386; 10.4269/ajtmh.1999.60.377 (1999).

Ndao, M. *et al*. *Trypanosoma cruzi* infection of squirrel monkeys: comparison of blood smear examination, commercial enzyme-linked immunosorbent assay, and polymerase chain reaction analysis as screening tests for evaluation of monkey-related injuries. *Comp. Med.* **50**, 658-665 (2000).

Schijman, A.G. *et al*. Differential detection of *Blastocrithidia triatomae* and *Trypanosoma cruzi* by amplification of 24Sα ribosomal RNA genes in faeces of sylvatic triatomine species from rural northwestern Argentina. *Acta Trop.* **99**, 50-54; 10.1016/j.actatropica.2006.06.010 (2006).

## Text S2 – Testing a small subset of vectors with a high-performance qPCR may help enhance *Trypanosoma cruzi* surveillance

We aim at getting estimates of the sensitivity and specificity of *Trypanosoma cruzi* detection through optical microscopy (OM) in entomological-parasitological routine surveillance (EPRS).

We postulate that this could be done by testing a random sample of the bugs with a high-performance qPCR in a reference/collaborating lab (at a state-level health agency or a University). In particular, we postulate here that testing a random subset of just 96 bugs with a single qPCR may suffice to estimate EPRS performance parameters with acceptable accuracy and precision. To test this, we ran a model as follows:

- *Data*
    - Retain only three results: OM slides (fresh and stained) read in EPRS + qPCR (first replicate only)
    - Draw 96 bugs at random using the R command `cbind(sample(1:841, 96, replace=FALSE))`; we chose *n* = 96 because qPCRs are run in 96-well

plates, so this number will correspond to a single plate for every ~850 bugs checked for infection in EPRS (the procedure selected bugs 1, 2, 7, 12, 26, 30, 42, 62, 64, 71, 77, 87, 88, 104, 106, 116, 119, 121, 123, 128, 149, 154, 162, 165, 173, 175, 184, 187, 190, 196, 198, 218, 228, 244, 245, 257, 258, 290, 303, 328, 332, 336, 365, 371, 377, 379, 384, 395, 397, 398, 399, 402, 409, 416, 418, 424, 426, 439, 444, 447, 448, 450, 467, 472, 475, 476, 493, 502, 505, 509, 538, 544, 558, 560, 562, 576, 599, 630, 631, 649, 659, 661, 675, 677, 686, 689, 692, 729, 732, 734, 735, 756, 793, 803, 805, and 834)

- Check that all selected bugs have at least one of the three results (at least one EPRS slide or the qPCR); the 96 bugs above matched this condition
- Recode all qPCR results to
  - "2" for the 96 randomly selected bugs (i.e., assume that qPCR detections are unambiguous, based on the specificity estimate from the paper [*Sp* = 0.999, or ~ 1.0])
  - "-" for the remaining bugs (i.e., turn them into 'missing' data)

- *Model*
  - Set EPRS sensitivity and specificity to be equal for both slides (i.e., estimate just a mean sensitivity and a mean specificity for EPRS slide-reading)
  - Fix the sensitivity of qPCR at 0.99 (slightly lower than estimated in the paper, where *Se* = 0.997)
  - Fix the specificity of qPCR at 1.0 (in the paper, *Sp* = 0.999)
  - Set *b* = 0.0 for slides (all detections are ambiguous, so none is coded as certain) and *b* = 1.0 for qPCR (when a qPCR detection occurs, it is coded as "2" with probability 1.0)
- Run the model and get estimates mean EPRS slide-reading detection parameters:
  - Sensitivity = 71.4% (CI 63.2–78.4%)
  - Specificity = 99.7% (CI 98.4–99.9%)

These results suggest that, despite a modest upward bias, testing about 1% of the bugs with a high-performance qPCR would provide fairly reliable estimates of slide-reading performance in EPRS. By doing this once a year, one could get a time-specific

‘correction factor’ for EPRS results from a given area (e.g., a state or municipality). We see this as a potentially important improvement (particularly regarding sensitivity) over the typical stance of ignoring the problem by assuming that detection is always perfect.

**Table S1.** The focal model: structure and numerical estimates back-transformed to the probability scale

| Test/Triatomine species | Parameter | Estimate | CI lower | CI upper |
|---|---|---|---|---|
| Test (performance) | | | | |
| Fresh slide EPRS | $Se_1$ | 0.245 | 0.202 | 0.295 |
| | $Sp_1$ | 0.926 | 0.898 | 0.946 |
| | $b_1$ | 0* | - | - |
| Fresh slide UnB | $Se_2$ | 0.143 | 0.102 | 0.199 |
| | $Sp_2$ | 0.931 | 0.882 | 0.960 |
| | $b_2$ | 0* | - | - |
| Stained slide EPRS | $Se_3$ | 0.507 | 0.424 | 0.590 |
| | $Sp_3$ | 0.928 | 0.890 | 0.954 |
| | $b_3$ | 0* | - | - |
| Stained slide UnB | $Se_4$ | 0.226 | 0.183 | 0.276 |
| | $Sp_4$ | 0.971 | 0.951 | 0.983 |
| | $b_4$ | 0.761 | 0.648 | 0.846 |
| cPCR rDNA-24Sα | $Se_5$ | 0.560 | 0.507 | 0.611 |
| | $Sp_5$ | 1.0 | ** | ** |
| | $b_5$ | 0* | - | - |
| qPCR nDNA-sat | $Se_6$ | 0.997 | 0.982 | 0.999 |
| | $Sp_6$ | 0.999 | 0.950 | 1.000 |
| | $b_6$ | 0* | | |
| Species (infection) | | | | |
| *Panstrongylus megistus* | $\Psi_1$ | 0.447 | 0.399 | 0.495 |
| *Triatoma sordida* | $\Psi_2$ | 0.326 | 0.273 | 0.384 |
| *Rhodnius neglectus* | $\Psi_3$ | 0.446 | 0.348 | 0.548 |
| *Triatoma pseudomaculata* | $\Psi_4$ | 0.667 | 0.518 | 0.788 |
| *Panstrongylus geniculatus* | $\Psi_5$ | 0.188 | 0.062 | 0.448 |

CI lower and CI upper, lower and upper limits of the 95% confidence interval
EPRS, entomological-parasitological routine surveillance; UnB, university of Brasília; cPCR, conventional PCR (of ribosomal DNA, rDNA-24Sα); qPCR, real-time PCR (of nuclear satellite DNA, nDNA-sat)
*Se*, sensitivity; *Sp*, specificity; *b*, probability that a detection is classified as certain, given that the bug was infected and a detection occurred; Ψ, species-specific probability of infection (or infection frequency)
*Fixed values; **CI could not be estimated (see main text)
